# A Case of Late-Onset Group B Streptococcus Serotype Ⅰb Meningitis With Absence of Pleocytosis at the Initial Cerebrospinal Fluid Analysis

**DOI:** 10.7759/cureus.31138

**Published:** 2022-11-05

**Authors:** Rie Chida, Shinichiro Morichi, Yusuke Watanabe, Gaku Yamanaka

**Affiliations:** 1 Department of Pediatrics and Adolescent Medicine, Tokyo Medical University, Tokyo, JPN

**Keywords:** infantile infection, group b streptococcus (gbs), absent of pleocytosis, late-onset group b streptococcus meningitis, bacterial meningitis

## Abstract

Bacterial meningitis in infants is a life-threatening illness that survives significant neurological sequelae that remain in survivors. The current diagnostic gold standard for meningitis is bacterial isolation from culture or molecular diagnostics in the cerebrospinal fluid. The decision for antibiotics therapy before bacterial detection is made on microscopic and biochemical findings in the cerebrospinal fluid, however, some patient shows no microscopic finding and pleocytosis at the initial cerebrospinal fluid analysis. Herein, we report a case of late-onset group B *Streptococcus* serotype Ib meningitis that could be introduced with timelier antibiotic therapy even in the absence of pleocytosis without the detection of bacteria on smear at the initial CSF analysis.

## Introduction

Bacterial meningitis in infants is a life-threatening illness that survives significant neurological sequelae that remain in survivors. The clinical picture of acute bacterial meningitis mainly depends on the patient’s age, and the classic manifestations noted in older children are rarely present in infants [1]. Classic symptoms of meningitis in children usually begin with fever, chills, vomiting, photophobia, and severe headache, however, younger children show subtle and atypical signs and symptoms [1]. The current gold standard for the diagnosis of meningitis is the identification of bacteria by culture or molecular diagnostics in cerebrospinal fluid (CSF) [2]. Until these results become available, the decision on whether or not to treat the child with antibiotics is often made on microscopic and biochemical findings in the CSF [2]. However, 2.7-25% of pediatric meningitis patients showed no pleocytosis at the initial CSF analysis because of intravenous antibiotic administration prior to lumbar puncture, insufficient CSF draw, development of meningitis secondary to another source, immunocompromised state, fulminant course of meningitis, and insufficient meningeal inflammation [2-4]. Atypical laboratory findings are at a higher risk of being missed, face a delay in receiving appropriate treatments, and increase the risk of a poor prognosis [4].

A procalcitonin (PCT) assay is an accurate screening test for identifying bacteremia and bacterial meningitis in febrile infants to 7-91 days old as compared to being moderately useful for severe bacterial infections, including urinary tract infections and bacterial gastroenteritis [5]. Therefore, the PCT assay can be a supplemental diagnostic method, combined with careful analysis of the case history, physical examination, and appropriate tests [5].

Herein, we report a case of late-onset group B Streptococcus (GBS) serotype Ib meningitis whose first symptoms were fever and diarrhea without pleocytosis and no bacteria detection on smear at the initial CSF analysis. However, it was successfully diagnosed by close observation of the patient and a serum PCT assay.

## Case presentation

A two-month-old Japanese boy was referred to our hospital due to persistent fever. He developed a fever the day before his presentation and received a medical consultation at night. However, he was not admitted, and based on his appearance, he likely had a viral infection. His mother complained that he had a fever and an increased frequency of stools compared to the usual. He was born by spontaneous vaginal delivery to a mother with negative vaginal GBS culture at term. The patient had a birth weight of 3290 g. He had a history of admission to the neonatal intensive care unit (NICU) due to vomiting and underwent outpatient laser treatment for ectopic Mongolian spots.

**Figure 1:**
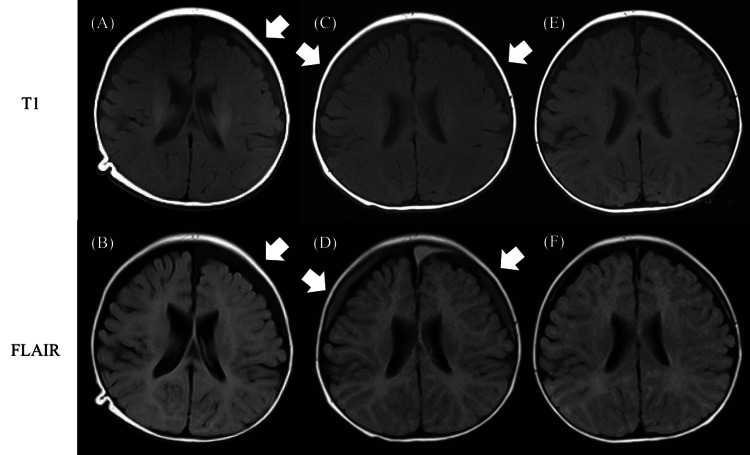
Brain magnetic resonance imaging of the patient (A) and (B) day 17 after admission. Subdural effusion (arrow) was observed in the left frontal lobe. (C) and (D) day 51 showed subdural effusion expanded bilaterally. (E) and (F) day 150 showed that the subdural effusion had disappeared.

On admission, his body temperature, blood pressure, heart rate, respiratory rate, and oxygen saturation were 39.2 °C, 84/42 mmHg, 181 beats/min, 42 beats/min, and 99% in room air, respectively. He was irritable, and a physical examination showed a flat anterior fontanelle without neck rigidity. Full sepsis workup and fecal rapid test for norovirus, rotavirus, and adenovirus were performed. The results showed a white blood cell (WBC) count of 7,800 cells/µL (reference range: 3300 - 8600 /μL) and C-reactive protein (CRP) of 11.5 mg/dL (reference range: < 0.3 mg/dL) in the blood, 5-9 WBCs per high-power field (reference range: < 4 /HPF) without bacterial detection on urine smear, positive result on fecal adenovirus rapid test, WBC count of 1 cells/mm^3^ (polymorphonuclear cells 75 %), glucose 50 mg/dL (reference range: 50 - 75 mg/dL), and total protein of 27 mg/dL (reference range: 10 - 40 mg/dL) in the CSF without bacterial detection on smear. We initially considered the most likely diagnosis of pyelonephritis or adenovirus gastroenteritis because of his symptoms and the examination results. However, the patient’s heart rate increased to 200 beats/min a few hours after the examination, even when an extracellular fluid infusion was initiated. Serum PCT was additionally measured, and it was markedly elevated at 44.4 ng/mL (reference range: < 0.5 ng/mL). We retracted our initial diagnosis and started empiric antimicrobial therapy with amoxicillin and cefotaxime for suspected bacterial meningitis. The baby vomited at night, 36 hours after the onset of fever. On day 2 after hospitalization, his fever persisted with increased irritability, and he started groaning. GBS was isolated from the CSF culture but not from the blood culture collected on admission. The microbiology department determined the serotype of GBS isolated from the patient’s CSF culture to determine its virulence and identified it as GBS serotype Ib. The diagnosis of late-onset GBS meningitis was confirmed. Additional therapy with dexamethasone every six hours for late-onset GBS meningitis and intravenous immunoglobulin for invasive bacterial infection was initiated. On day 3, his persistent fever resolved. We repeated the lumbar puncture and the second CSF analysis showed WBC of 997 cells/mm^3^, glucose 64 mg/dL, and total protein 121 mg/dL. Antibiotic susceptibility testing revealed that the organism was sensitive to amoxicillin; therefore, we discontinued the administration of cefotaxime. The third CSF analysis was performed on day 11 to evaluate whether to cease antibiotic therapy, which revealed decreased glucose of 34 mg/dL even without bacterial detection in the second CSF culture. Brain magnetic resonance imaging revealed subdural effusion in the left frontal lobe on day 17 (Figure 1).

We suspected that the inflammation of the meninges persisted even without the detection of bacteria in the consecutive CSF cultures because the CSF glucose level decreased 11 days after the onset of treatment. CSF glucose levels were found to be elevated to normal 30 days after admission; therefore, antibiotic therapy was terminated on day 35. The recurrence of symptoms was not observed after the cessation of therapy. To confirm neurological complications, fundoscopy, auditory brainstem response, electroencephalogram, and MR angiography were performed, and all examinations were normal. We also checked his serum immunoglobulins, immunoglobulin G (IgG) blood levels, and B-cell subsets by flow cytometry to identify the underlying disease. All results were within the normal range, thus excluding any immunodeficiencies. On day 54 after admission, all evaluations on the patient’s condition were completed, and he was finally discharged. He is now two years old and continuing outpatient follow-up. The subdural effusion disappeared at seven months old, and he has displayed normal development without any neurological complications.

## Discussion

Herein, we report a case of late-onset group B Streptococcus (GBS) serotype Ib meningitis whose first symptoms were fever and diarrhea without pleocytosis and no bacteria detection on smear at the initial CSF analysis; however, it was successfully diagnosed by patient closed observation and serum PCT assay. In addition to atypical symptoms and initial CSF analysis, results of urinalysis and adenovirus fecal rapid test which coincided with the patient’s symptoms made the decision for the diagnosis and treatment of bacterial meningitis more difficult.

Previous studies reported 2.7-25% of pediatric meningitis cases showed no pleocytosis at the initial CSF analysis [2-4]. Intravenous antibiotic administration prior to lumbar puncture, insufficient CSF draw, development of meningitis secondary to another source, immunocompromised state, and a fulminant course of meningitis lead to an absence of pleocytosis at the initial CSF analysis [4]. However, most patients undergo lumbar puncture in less than or equal to 24 hours of the onset of fever; therefore, there is a possibility that the meninges are not yet sufficiently inflamed to lead to pleocytosis [2]. The potential benefit of a second lumbar puncture, especially in the absence of pleocytosis at the initial CSF analysis and suspected bacterial meningitis, has been discussed [2]. It has also been stated that low CSF WBC count correlates with poor outcomes in pediatric patients with bacterial meningitis because these patients are at high risk of being misdiagnosed or treated late [4].

We initially started empiric treatment for pyelonephritis or adenovirus gastroenteritis because of the results of laboratory exams, however, the unstable patient’s heart rate increased to 200 beats/min even after the extracellular fluid infusion was initiated during laboratory exams, and this made us reconsider the initial diagnosis. Invasive bacterial infection was highly suspected from the course of the patient's illness. Therefore, to rule out acute pyelonephritis and adenovirus gastroenteritis, the patient’s serum PCT assay was performed. PCT is an accurate screening test for identifying bacteremia and bacterial meningitis in febrile infants between 7 and 91 days old [5]. PCT levels are markedly elevated within 4 hours and peak between 12-48 hours after the onset of systemic bacterial infection [6]. The cut-off value of serum PCT used to rule out invasive bacterial infections in infants aged 7-91 days admitted for fever in a previous study is 0.3 ng/mL [5]. For adenovirus infections, the levels of serum PCT level range from 0.04-5.67 ng/mL (median 1.14 ng/mL) [7]. The level of serum PCT that distinguishes between invasive bacterial infections and adenovirus infections is vague; however, in our patient, PCT was markedly elevated; therefore, an invasive bacterial infection was more probable than adenovirus gastroenteritis or a urinary tract infection.

Although the patient showed atypical symptoms and laboratory exams, close observation, especially of the patient’s vital signs and additional serum PCT assay made it possible for us to make a timelier diagnosis of bacterial meningitis, and the patient had no neurological complications at the time of being discharged and even normal development was observed during the outpatient follow-up period. We considered the patient’s meninges were not yet sufficiently inflamed at the time of the initial lumbar puncture because there was no antibiotic use prior to the lumbar puncture, he was immunologically normal, and vomiting was observed 36 hours after the fever onset.

GBS disease is now the third most common cause of bacterial meningitis in children after Streptococcus pneumoniae and Neisseria meningitidis infection, and the leading cause of neonatal bacterial meningitis [8]. Recognized risk factors for mortality are prematurity, birth weight, and seizure [8]. GBS serotype Ib is the third most prevalent (5.3%) serotype that causes late-onset GBS infection worldwide [9]. The GBS serotype Ⅲ with CC17 has been known to be more virulent and enhances its invasiveness in neonates, however, GBS serotype Ib has been less known; only one case report described a clinical picture of GBS serotype Ib meningitis [8]. In this previously reported case, a 54-day-old infant born extremely preterm was admitted to the neonatal intensive care unit (NICU) and died due to GBS serotype Ib bacteremia and meningitis five hours after the onset of tachycardia following refractory apnea and bradycardia [10]. Our patient was a term infant with a history of NICU admission who developed fever, diarrhea, irritability, and vomiting over 36 hours after the onset of fever. Our patient survived GBS serotype Ib meningitis without any complications. Disease progression and outcomes were dramatically different between the two cases. We cannot identify the factors related to disease progression and outcomes because only one case report was available prior to this one. The difference between the two patients was gestational age. We evaluated whether our patient had underlying immune diseases that are a risk factor for invasive bacterial infection, and all the immunology examinations were normal. We also attempted to identify the source of the infection. Blood, CSF, throat, urine, and fecal samples were collected from our patient. Additionally, a throat swab was collected from both parents, and breast milk was collected from the mother for culture. GBS serotype Ib was isolated only from the first CSF culture from the patient; therefore, we were unable to identify the source of the infection. More research is required to clarify the clinical picture of GBS serotype Ib meningitis.

The limitations of our case report are the lower number of references and no other infantile cases of bacterial meningitis with the absence of pleocytosis at the initial CSF analysis available to compare to this case.

## Conclusions

Physicians should be aware that the absence of pleocytosis at the initial CSF analysis does not exclude bacterial meningitis. If laboratory examination results cannot explain the patient's status, medical staff must keep close observation and add supplemental exams, such as serum PCT assay, to avoid missing bacterial meningitis. More research is required for both bacterial meningitis with the absence of pleocytosis at the initial CSF analysis and GBS serotype Ib meningitis to clarify useful supplemental exams and clinical pictures for timely diagnosis.
